# Intraoperative fracture of phacoemulsification sleeve

**DOI:** 10.1186/1471-2415-10-29

**Published:** 2010-11-30

**Authors:** Jennifer WH Shum, Keith SK Chan, David Wong, Kenneth KW Li

**Affiliations:** 1 Department of Ophthalmology, United Christian Hospital, Kwun Tong, Kowloon, HKSAR, China; 2 Department of Ophthalmology, Queen Mary Hospital, Pokfulam, HKSAR, China; 3 Eye Institute, LKS Faculty of Medicine, The University of Hong Kong, HKSAR, China

## Abstract

**Background:**

We describe a case of intraoperative fracture of phacoemulsification sleeve during phacoemulsification surgery.

**Case presentation:**

Phacoemulsification surgery was performed in the left eye of a 58-year-old lady with grade II nuclear sclerosis & grade I cortical cataract. Towards the end of quadrant removal, there was anterior chamber instability with impaired followability of nuclear fragments. The distal part of the fractured sleeve remained inside the anterior chamber upon removal of the phacoemulsification probe. The retained sleeve was retrieved with a pair of forceps through the corneal incision site, which did not require widening. There was no missing fragments retained intraocularly and the patient had an uneventful recovery with vision of 20/25 at three months post-operatively.

**Conclusion:**

Phacoemulsification sleeve fracture is an uncommon complication. With early identification of this condition and proper management, major complications can be avoided.

## Background

Instruments breakage during phacoemulsification surgeries can have detrimental effect on the eye. Common breakages can involve the phacoemulsification tips and second instruments. Intraoperative fracture of phacoemulsification sleeve occurs infrequently. We hereby report a case of intraoperative phacoemulsification sleeve fracture. The early signs of this condition are described and surgical management of this situation is discussed.

## Case presentation

A 58-year-old lady was clinically admitted for phacoemulsification surgery and intraocular lens (IOL) implantation in the left eye. She had the same surgery done to her right eye two years ago and later developed posterior capsule opacification requiring YAG posterior capsulotomy. Best-corrected visual acuity (BCVA) was 20/30 on the right eye and 20/100 on the left eye. Anterior segment examination revealed grade II nuclear sclerosis and grade I cortical cataract in the left eye. Intraocular pressure was within normal limits, and no other significant ocular abnormality was noted.

**Figure 1:**
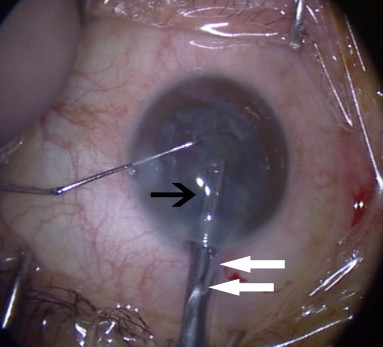
**Backflow of BSS from corneal incision**. Black arrow pointing to phacoemulsification sleeve. White arrow pointing to backflow of BSS.

Surgery was performed under topical anaesthesia with 2% xylocaine gel. A 2.75 mm superior corneal incision was created with keratome. Continuous curvilinear capsulorrhexis, hydrodissection and hydrodelineation were performed uneventfully. Phacoemulsification was then carried out using a standard 20-gauge phaco tip. Towards the end of segment removal, the anterior chamber (AC) was noted to be unstable. The followability and aspiration of nuclear fragments also appeared to be impaired. There was a sudden backflow of balanced salt solution (BSS) emerging from the corneal incision (Figure 1). The AC was still formed at this stage. The surgeon then attempted to withdraw the phacoemulsification probe out of the incision.

**Figure 2:**
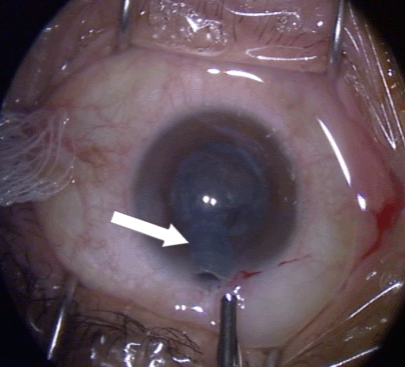
**Retained phacoemulsification sleeve**. White arrow showing retained phacoemulsification sleeve inside anterior chamber.

**Figure 3:**
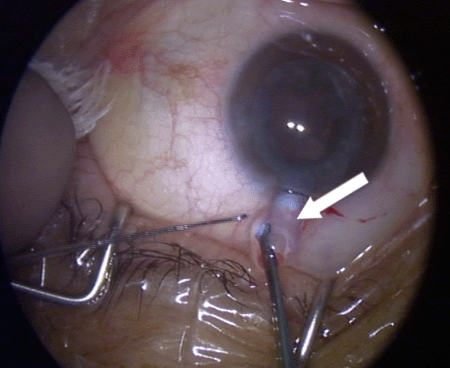
**Retrieval of phacoemulsification sleeve**. White arrow showing retrieval of phacoemulsification sleeve.

**Figure 4:**
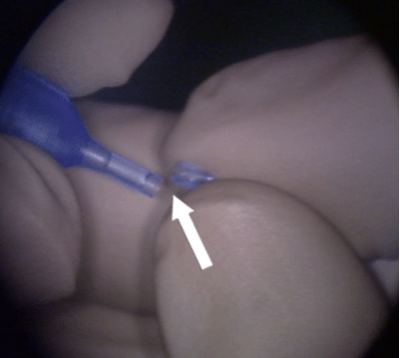
**Matching the broken phacoemulsification sleeve ends**. White arrow showing the matching ends of the broken phacoemulsification sleeve.

Resistance was encountered upon withdrawal of the phacoemulsification probe, with the sleeve apparently jammed against the cornea. This resistance suddenly gave way upon increased traction with resultant severance of the silicon probe sleeve, the distal part of which remained inside the anterior chamber (Figure 2). Viscoelastic was used to reform the AC and the retained sleeve was retrieved with a pair of fine non-tooth forceps through the corneal incision site, which did not require widening (Figure 3). The anterior chamber was checked for any possible retained smaller fragments of the sleeve and none were noted. The two broken pieces of sleeve were examined carefully. The broken ends matched and there were no missing fragments (Figure 4). A thorough search for any potential fragments using the operating microscope did not reveal anything. The operation, including IOL implantation, was completed uneventfully and the incision was closed with one 10-O nylon suture. She had uneventful recovery and at three-month follow-up, patient recovery was satisfactory and her BCVA of the left eye was 20/25.

## Discussion

Breakage of instruments during phacoemulsification can lead to retained foreign bodies in the AC. These commonly arise from second instrument tips, irrigation tubing and the phacoemulsification probe [1-5]. These retained fragments can potentially lead to increase in postoperative inflammation and damage to the intraocular tissue. Fracture of the phacoemulsification sleeve occurs infrequently. There was only one report in the literature in which the retained foreign body arose from the inner lining of the phacoemulsification sleeve [6]. It was fortunate that the fracture of the phacoemulsification sleeve did not result in multiple fragments and therefore there was no retained foreign object in the present case.

Fracture of the phacoemulsification sleeve could give rise to other complications apart from retained foreign body. Therefore early identification of this condition is important.

**Figure 5:**
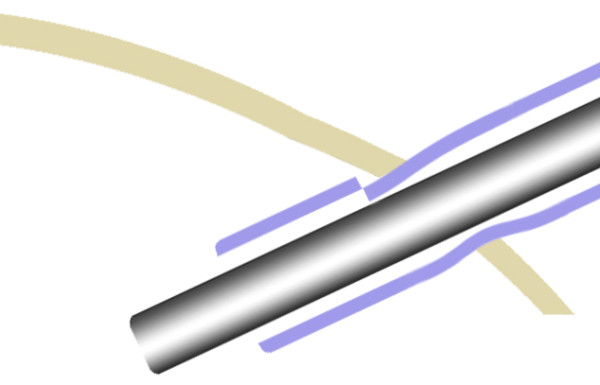
**Illustration showing jammed distal phacoemulsification sleeve**. The jammed distal phacoemulsification sleeve is larger in diameter, giving rise to resistance on withdrawal of sleeve.

We believe that initially there was a small partial-circumferential fracture of the phacoemulsification sleeve. Stress on the phacoemulsification probe during surgery progressively extended the fracture. In the beginning, the structural turgidity was not yet impaired. This explains the smooth insertion of the probe on commencement of surgery, and the uneventful chopping. At some point, the fracture size was significant enough as to impair the structural turgidity of the sleeve. We believe that the fractured distal part of the sleeve was jammed in the incision (Figure 5). Since the sleeve was elastic, it was possible that the distal part of the sleeve was still in the AC and the break was exposed outside of the incision. This could explain the initial backflow of BSS. When the surgeon withdrew the phacoemulsification probe, the break was completed causing complete severance. The above mechanism would also explain the backflow of BSS and resistance encountered on withdrawing the probe. We reckon that the fracture site was within AC when the backflow of BSS was encountered because the AC, however unstable, was still formed. Moreover, resistance was encountered on withdrawal of the probe.

Another early sign we noted was the suboptimal followability and aspiration during segment removal. The design of placing the irrigation port at the sides of the phacoemulsification sleeve is intended to create an ideal laminar flow inside the anterior chamber [7]. We hypothesize that the fracture of the sleeve resulted in turbulance, eddy currents and thus impaired followability. Furthermore, with reduced AC pressure, the effective pressure gradient that can be generated at the phacoemulsification needle tip is also decreased. For example: bottle height at 80 cm translates into an AC pressure of approximately 60 mmHg. At a vacuum of 100 mg generated by the pump, AC pressure would account for 44% of the pressure gradient.

Using brute force to withdraw the probe carries the risk of lacerating the cornea incision. Fortunately in our case, the distal part of the sleeve broke off. If much resistance was met upon withdrawal of the probe, one should first stop the infusion of BSS and reform the AC with viscoelastic through the side-port. If there is resistance on withdrawing the phacoemulsification probe, one can try rotating the probe first, in order to reverse the jam, before attempting to withdraw it again. Excessive force should be avoided to prevent complete severance of the sleeve.

As to how the fracture of the sleeve occurred, we have no clear answer to offer. It could have been due to a manufacturing oversight, or due to trauma induced when the scrub nurse applied the phacoemulsification sleeve on the probe. However, we would like to touch on the advent of microincision cataract surgery (MICS) and the ensuing development of microinstruments. As technology further advances, we are now able to use minimal ultrasound energy for phacoemulsification using smaller probes through incisions as small as 1.8 mm. Thus, the traditional role of the phacoemulsification sleeve as an insulation against heat and friction has become theoretically less important, and the phacoemulsification sleeve diameters have been incrementally decreasing with many less than 0.40 mm in thickness. One should also bear in mind that the phacoemulsification sleeve becomes increasingly delicate. It is anticipated that they may be more prone to damage, and manufacturing defect. We therefore advise careful inspection of all instruments before introducing them into the eye. This must include the phacoemulsification sleeve, which tends to be overlooked due to its complementary nature. In the future, in addition to examining the irrigation and aspiration of the phacoemulsification probe, we recommend sparing a few seconds to inspect the phacoemulsification sleeve for leaks or cracks.

## Conclusions

Phacoemulsification sleeve fracture is an uncommon complication. With early identification of this condition and proper management, major complications can be avoided.

## Competing interests

The authors declare that they have no competing interests.

## Authors' contributions

JS drafted the manuscript. KC correlated intraoperative findings and identification of signs. DW participated in discussion. KL provided guidance, supervision and final editing of manuscript. All authors have read and approved the final manuscript.

## Pre-publication history

The pre-publication history for this paper can be accessed here:

http://www.biomedcentral.com/1471-2415/10/29/prepub
